# Where differences resemble: sequence-feature analysis in curated databases of intrinsically disordered proteins

**DOI:** 10.1093/database/bay127

**Published:** 2018-12-14

**Authors:** Marco Necci, Damiano Piovesan, Silvio C E Tosatto

**Affiliations:** 1 Department of Biomedical Sciences, University of Padua, Via Ugo Bassi 58b, Padua, Italy; 2 Department of Agricultural Sciences, University of Udine, Via Palladio 8, Udine, Italy; 3 Fondazione Edmund Mach, Via Edmund Mach 1, San Michele all'Adige, Italy; 4 Institute of Neuroscience, National Research Council, Corso Stati Uniti 4, Padua, Italy

## Abstract

Intrinsic disorder (ID) in proteins is involved in crucial interactions in the living cell. As the importance of ID is increasingly recognized, so are detailed analyses aimed at its identification and characterization. An open question remains the existence of ID `flavors’ representing different sub-phenomena. Several databases collect manually curated examples of experimentally validated ID, focusing on apparently different aspects of this phenomenon. The recent update of MobiDB presented the opportunity to carry out an in-depth comparison of the content of these validated ID collections, namely DIBS, DisProt, IDEAL, MFIB, FuzDB, ELM and UniProt. In order to assess what is specific to different ID flavors, we analyzed relevant sequence-based features, such as amino acid composition, length, taxa and gene ontology terms, highlighting differences and similarities among datasets. Despite that, the majority of the considered features are not statistically different across databases, with the exception of ELM. FuzDB also shares half of its entries with DisProt. In general, different ID databases describe similar phenomena. DisProt, which is the largest database, better represents the entire spectrum of different disorder flavors and the corresponding sequence diversity.

## Introduction

After over a century, the paradigm stating that proteins need to fold into a stable structure to function was proven overly simplistic as many proteins were shown to be intrinsically disordered under physiological conditions (1–3). Intrinsically disordered proteins (IDPs) can both fold upon binding (4) and become unfolded following an interaction (5). They perform functions that are crucial to the life of organisms, taking advantage of these structural features (4, 6, 7). Detection of ID has posed a challenge in response to which experimental methods, either existing or purposely developed, have been employed (8, 9). Several features play an important role in describing ID. Amino acid frequencies of ID are enriched in charged and hydrophilic amino acids and depleted of hydrophobic ones (10). Different molecular phenomena like phase separation or fuzziness have also been associated to particular amino acid propensities (11). Length of intrinsically disordered regions (IDRs) affects their behavior in function (12) and evolution (13), with long segments being more likely functionally relevant and mostly involved in molecular recognition (12). Charge distribution of ID influences its structural propensities, either favoring a more relaxed (random coil) or more compact (molten globule) conformation (10). Low sequence complexity correlates with ID, since LC regions were found more frequently in proteins with ID than in a set of structured proteins (14).

Results from a fraction of ID experiments have been manually curated in specialized databases. Historically, the first ID database was DisProt (15), released in 2005 thanks to the rapid growth of experimental data describing ID. It was mostly abandoned for some years until the release of a completely re-annotated and expanded database (16). IDEAL (17) is a collection of experimentally verified IDRs, with special attention to functional IDRs. Together, both IDEAL and DisProt are the largest ID databases. More recently, three new databases focusing on different aspects, or flavors, of ID were released. DIBS (18) is a systematic analysis of the structural/functional principles underlying the assembly of complexes between IDPs and their globular partners. MFIB (19) investigates the principles underlying protein complexes formed exclusively by IDP partners. FuzDB (20) focuses on the functional impact of the seemingly stand-alone phenomenon of fuzzy protein complexes, where ID is maintained upon interacting with partners. In addition to these databases, focusing entirely on ID, some experimentally validated ID annotations can be obtained from ELM (21) and UniProt (22). ELM annotates short functional sites in eukaryotic protein sequences that are mostly found in IDRs (23). Finally, among the many curated annotations in UniProt, it also contains some experimentally validated IDRs. All these databases apparently collect different ID flavors. The degree to which this assertion holds is however an open question. Inclusion of all of the above experimental sources in the newest version of MobiDB (24), a centralized resource for annotations of ID, gave us the opportunity to directly compare them.

In this work, we wondered whether different ID flavors as defined in the specialized databases can be easily discriminated from each other and how different they really are. We investigated the differences and similarities between the different databases in terms of sequence features such as amino acid composition, length and charge distributions of IDRs and correlation of ID with low sequence complexity regions. Furthermore, we investigate whether different databases capture functions more frequently than others via a gene ontology (GO) term enrichment analysis. Our results suggest that ID databases are similar, yet differ in subtle ways, with DisProt being a collector of many different phenomena.

## Materials and methods

Entries from all databases are mapped to UniProt (22) when included in MobiDB 3.0 (25). Datasets were generated by querying the MobiDB 3.0 database directly for annotations coming from different databases, namely DisProt (16), UniProt (22), ELM (21), IDEAL (17), MFIB (19), DIBS (18) and FuzDB (20). IDEAL, which in MobiDB only includes `protean segments’ was integrated to cover all manually annotated annotations. MobiDB 3.0 uses a concatenation strategy, where overlapping annotations are flattened to a series of adjacent annotations. In this analysis, adjacent ID annotations are merged into unique regions (Supplementary Figure S1). All the calculations were made by considering these merged IDRs with the only exceptions of dataset intersection, taxonomy and functional enrichment, which are calculated considering the full-length proteins. The intersection is calculated considering UniProt accessions and visualized as Venn diagram generated using Jvenn (26). Protein taxa of origin are obtained from the MobiDB 3.0 annotation, which in turn uses the UniProt full taxonomy information. GO terms are taken from UniProt including predicted ones, i.e. those with inferred from electronic annotation evidence codes. Region overlap between datasets is calculated counting overlapping ID residues. Unique ID residues are those not overlapping with any other database. Amino acid frequencies are the number of a given amino acid in a dataset divided by the total number of amino acids in the same dataset. Amino acid enrichment and depletion for each database is measured normalizing (subtracting and then dividing) by TrEMBL absolute frequencies. The TrEMBL amino acid frequencies are obtained from the TrEMBL statistics webpage (amino acid distribution statistics section). Hierarchical clustering of amino acid enrichment (and row frequencies) is based on the Euclidean distance (d) between two vectors (u and v) with V as the variance vector:

$$ d=\sqrt{\sum \frac{{\left({u}_i-{v}_i\right)}^2}{V\left({x}_i\right)}}. $$

The classification of IDRs into classes as proposed by Das and Pappu (27, 28) is performed calculating the two values *f_+_* and *f_-_*, the fraction of positively charged and negatively charged residues, for each IDR in a dataset. Low complexity (LC) regions were obtained by querying MobiDB 3.0 for SEG (29) predictions. LC content of IDRs is computed for each dataset as the fraction of residues in an IDR predicted by SEG software as LC, against the total IDR length. GO term (30) enrichment values (E) were calculated as

$$ E={\log}_{10}\frac{A}{C}-{\log}_{10}\frac{B}{D},  $$

where A is the cluster count of the GO term GO_i_, B the background count for GO_i_, C the number of GO terms in the cluster different from GO_i_ and D the number of GO terms in the background different from GO_i_. Prior to calculating E, a Fisher test *P*-value was applied to the contingency table for each GO term:

$$ p=\frac{\left(\genfrac{}{}{0pt}{}{A+B}{A}\right)\left(\genfrac{}{}{0pt}{}{C+D}{C}\right)}{\left(\genfrac{}{}{0pt}{}{n}{A+C}\right)}, $$

where $n=A+B+C+D$. Only terms with a two-tailed Bonferroni corrected *P*-value < 0.05 were included in the analysis. Furthermore, only terms with a minimum distance from the root of at most 3, 3 and 4 were considered for cellular component, biological process and molecular function, respectively.

Sequence redundancy was removed with CD-HIT (31) using global alignments with an identity cutoff of 90% to generate sequence clusters. The longest sequence in each cluster was used as the cluster representative. The effects of redundancy removal on amino acid frequencies were tested. Based on the similarity of results, we decided not to apply redundancy removal in subsequent analyses (data not shown).

The statistical significance is shown as a series of all-vs.-all comparisons in a square matrix where cells display correlation or significance values between database pairs. The Chi-square test is used for the taxa and Das and Pappu statistics, since these data contain different categories. For length and LC, significance is calculated using the student *t*-test. For all other statistics, significance is measured calculating the *P*-value of the Pearson correlation coefficient, with significance indicating correlated distributions. All tests and *P*-values are calculated using the Python SciPy library (URL: https://www.scipy.org/).

## Results

### Dataset composition and overlap

The simplest question is whether ID database annotations overlap. The number of proteins in different datasets (Table 1) ranges from 99 (FuzDB) to 1772 (ELM) with an average of approximately 637 proteins annotated per dataset. The IDEAL database contains more than 752 entries, 272 of which are annotated as `protean segments’ (17), a term describing regions undergoing coupled folding and binding. Since the number of non-overlapping regions in all datasets is higher than their number of proteins, all of them include some proteins with more than one annotation. In most datasets proteins are annotated on a single region, especially in UniProt and MFIB. On the other hand, DisProt and ELM have on average 1.5 and 1.4 regions per protein.

**Table 1:** Datasets composition. The number of IDPs, regions and amino acids in each dataset is shown

	Proteins	Regions	Residues
DIBS	465	514	12 770
DisProt	721	1087	81 632
ELM	1772	2579	19 325
FuzDB	99	140	8768
IDEAL	752	2783	47 315
MFIB	246	253	22 487
UniProt	406	424	38 224

**Figure 1:**
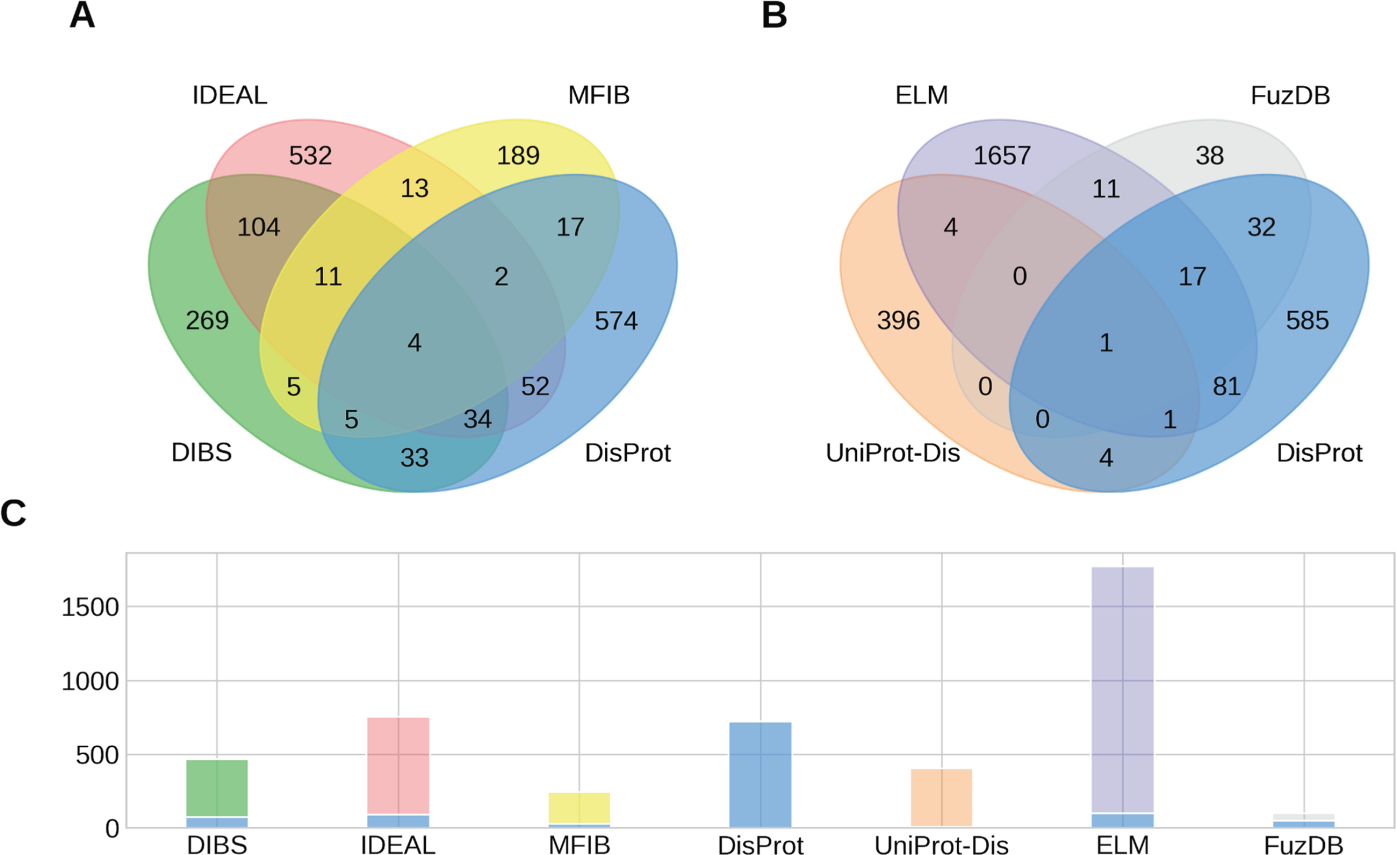
Overlap of IDPs annotated by multiple databases. (**A, B**) The Venn diagrams show the overlap between groups of four ID datasets. (**C**) The number of IDPs in each dataset is shown together with the fraction shared with DisProt (stacked blue bar).

**Figure 2:**
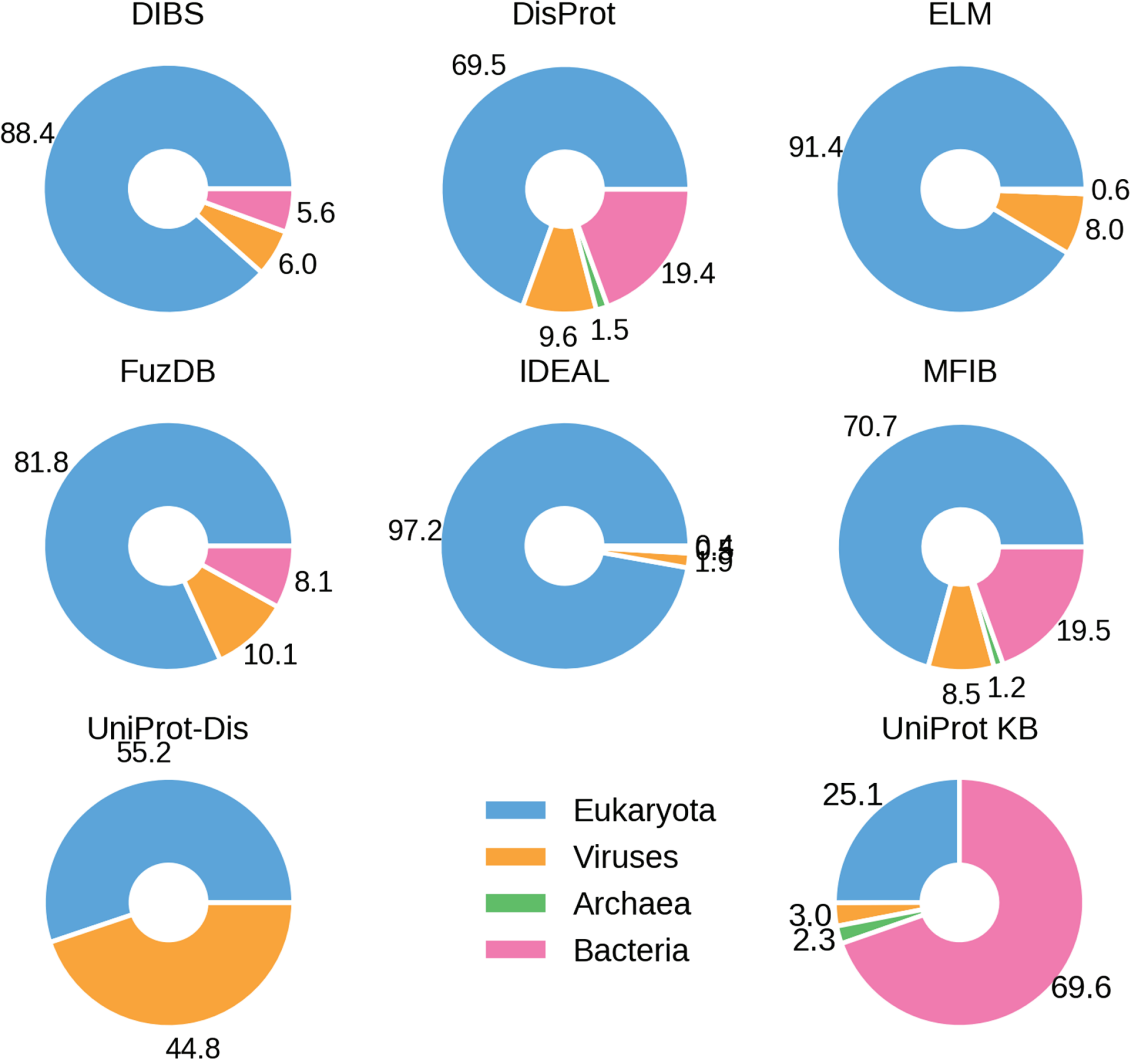
Percentage ID taxa of origin. Eukaryotes are shown in blue, viruses in pink and prokaryotes in orange. Archaea (in green) are mostly absent.

While the raw sum of proteins in the datasets is 4461, the union of all datasets counts 3621 proteins. This means that different datasets share 840 entries. Figure 1 shows the intersection of annotated proteins for the four databases with the largest overlap. Surprisingly, only four entries are annotated by all datasets. The count reaches 0 when considering the intersection of all datasets (data not shown). Despite the number of proteins annotated in the datasets being small compared to the number of known protein sequences, we would expect to find some relevant examples (e.g. p53 or alpha-synuclein) in all of them. Their absence may suggest the specialization of the datasets on different aspects of the wide range of ID phenomena. However, some datasets share more entries than others. The intersection between DisProt and DIBS counts 76 entries. This is expected, since the DIBS annotators use DisProt as an example pool to annotate disordered binding sites. The biggest intersection is between DIBS and IDEAL. Both annotate disordered binding sites undergoing coupled folding and binding. They share 153 entries, representing 20% of IDEAL and 33% of DIBS. Interestingly, DisProt shares 92 entries with IDEAL, but only less than half are also in DIBS. Significant overlaps can be found between DisProt and FuzDB (half of its entries), ELM (100 entries), DIBS (76) and IDEAL (92).With the notable exception of FuzDB, the proteins annotated for ID are therefore largely different. The overlap at the region level reflects the entries overlap, with DisProt, IDEAL and UniProt annotating the majority of total and unique ID residues (Supplementary Figure S2). FuzDB shares the 34% of its ID residues with DisProt. High overlap is also found between DIBS, DisProt and IDEAL (Supplementary Figure S3).

### Source organisms

The fraction of ID in different organisms gradually increases from bacteria to archaea and viruses, peaking in eukaryotes and especially in higher vertebrate groups, including mammals (12, 32, 33). We therefore investigated the species distribution in ID databases. The fraction of proteins belonging to one of the four domains of life (bacteria, archaea, eukaryota and viruses) in the different datasets confirms this trend (Figure 2). There are, however, some notable exceptions. DisProt and MFIB contain 19% of proteins coming from Bacteria, while in other datasets this number ranges from 0 to 8.1%. This is not due to sequence redundancy, since redundancy removal affected the analysis only marginally (data not shown). In general, DisProt and MFIB share a very similar profile. Similarly, DIBS and FuZDB are statistically not different (Supplementary Figure S4). On the other hand, UniProt has a higher fraction of viral proteins. Archaeal proteins are only marginally represented in DisProt, IDEAL and MFIB. As expected, the overall organism distribution of ID is largely maintained in the databases. An increase in bacterial proteins for DisProt and MFIBmight suggests a bias in the experimental systems used to test ID or might reflect a different strategy for the selection of the proteins to annotate.

**Figure 3:**
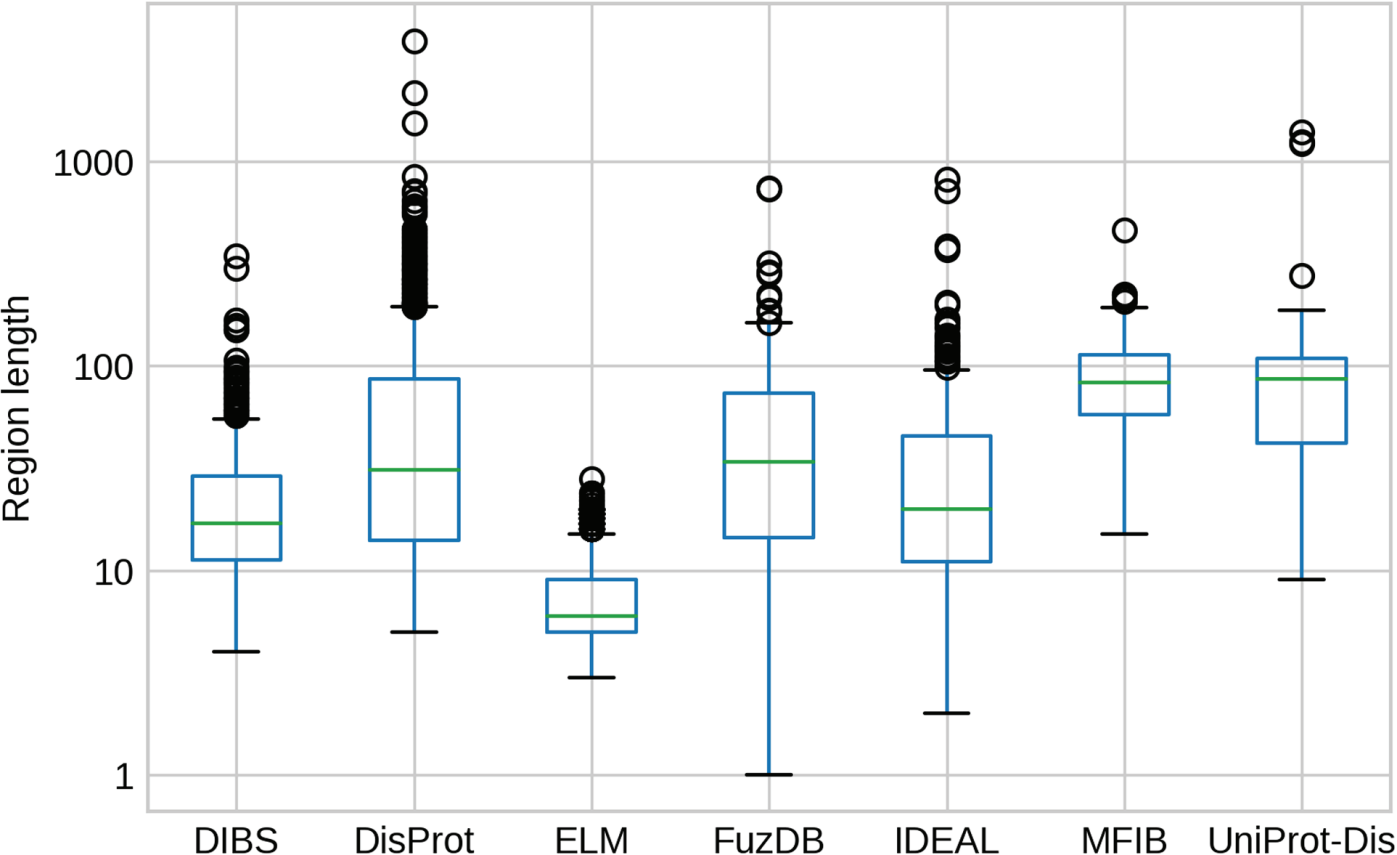
IDR length distributions. The *y*-axis is in logarithmic scale. Box plots of the region length distribution in each dataset. Whiskers represent the 25 and 75% of the data and outliers are shown as circles.

**Figure 4:**
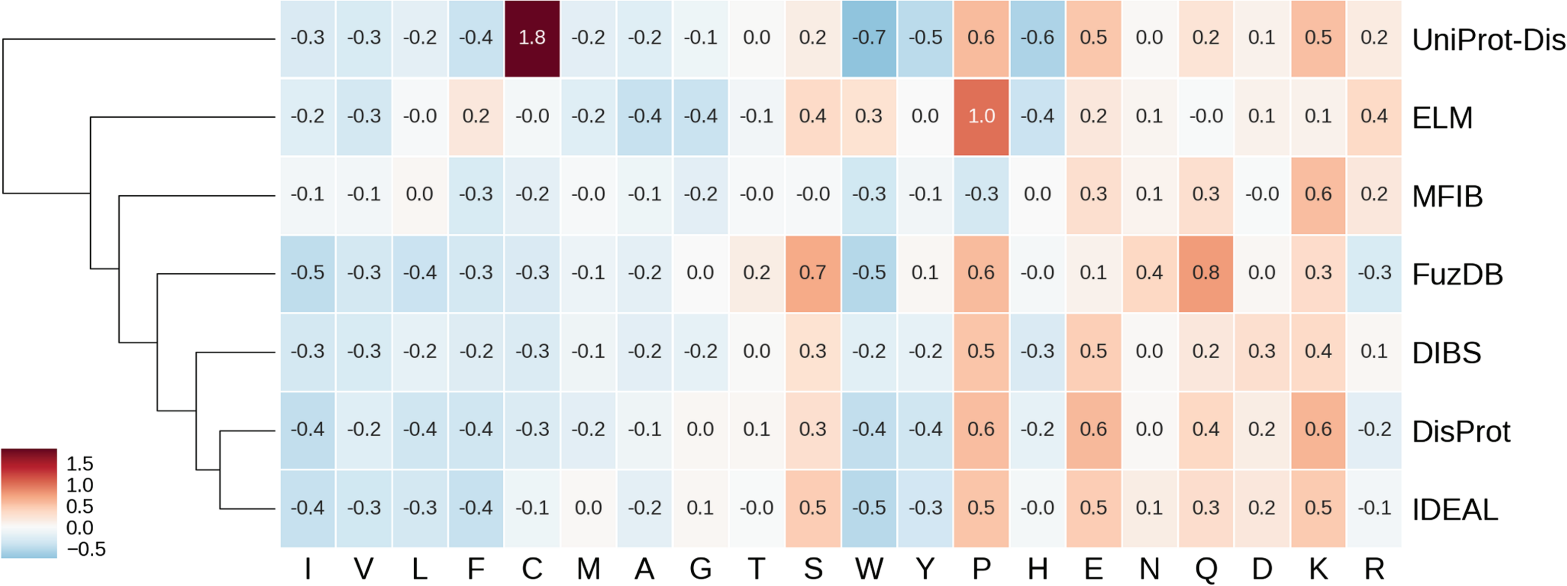
Hierarchically clustered heat map of amino acid enrichment. Clustering is based on Euclidean distance between frequency vectors. Each value represents the fold increase (red) or decrease (blue) compared to the TrEMBL amino acid distribution.

**Figure 5:**
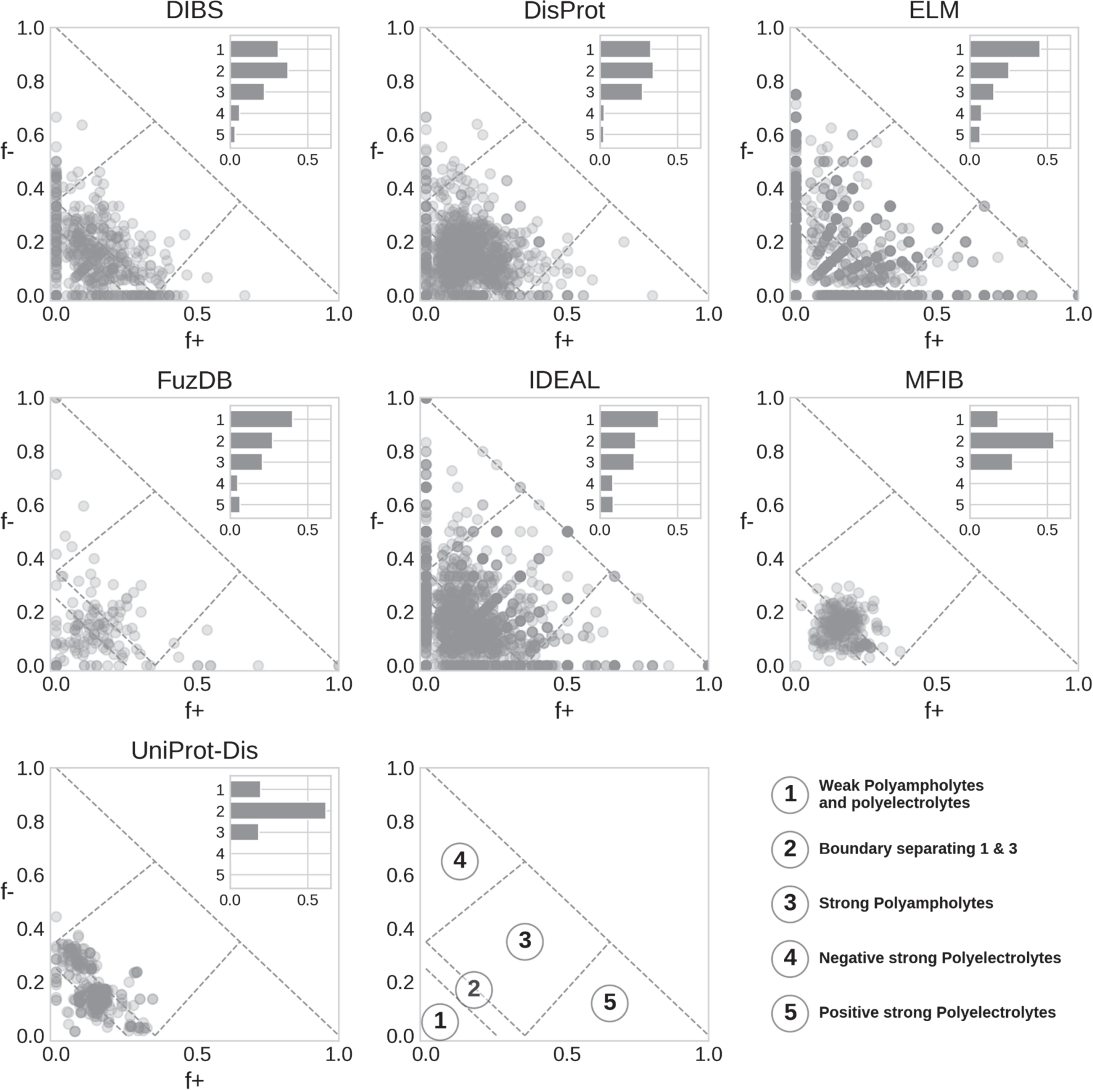
Das and Pappu classification of IDRs. Each marker represents a region, with the main Das and Pappu class numbers shown. The histogram is normalized by the total number of region in each database.

### Length distribution

The length of IDRs is known to discriminate their amino acid composition (34) and affect their behavior in both function (12) and evolution (13). We investigated whether different datasets display different IDR length distributions (Figure 3). As expected, ELM has the shortest regions, followed by DIBS, as it concentrates on short linear motifs. On the other hand, DisProt regions span from very short (at least five residues by design) to more than 1000 residues. For example the human BRCA1 (breast cancer type 1 susceptibility) protein contains a region spanning from residue 103 to 1646. However, the average length is higher for UniProt-Disorder and MFIB, possibly indicating that these datasets contain a greater fraction of long proteins. IDR distributions of FuzDB, IDEAL and DisProt are not significantly different from each other (Supplementary Figure S5), with the notable exception of a few FuzDB regions covering single residues. As suggested by similar shapes of the box plots, these three databases sample regions of similar length.

**Figure 6:**
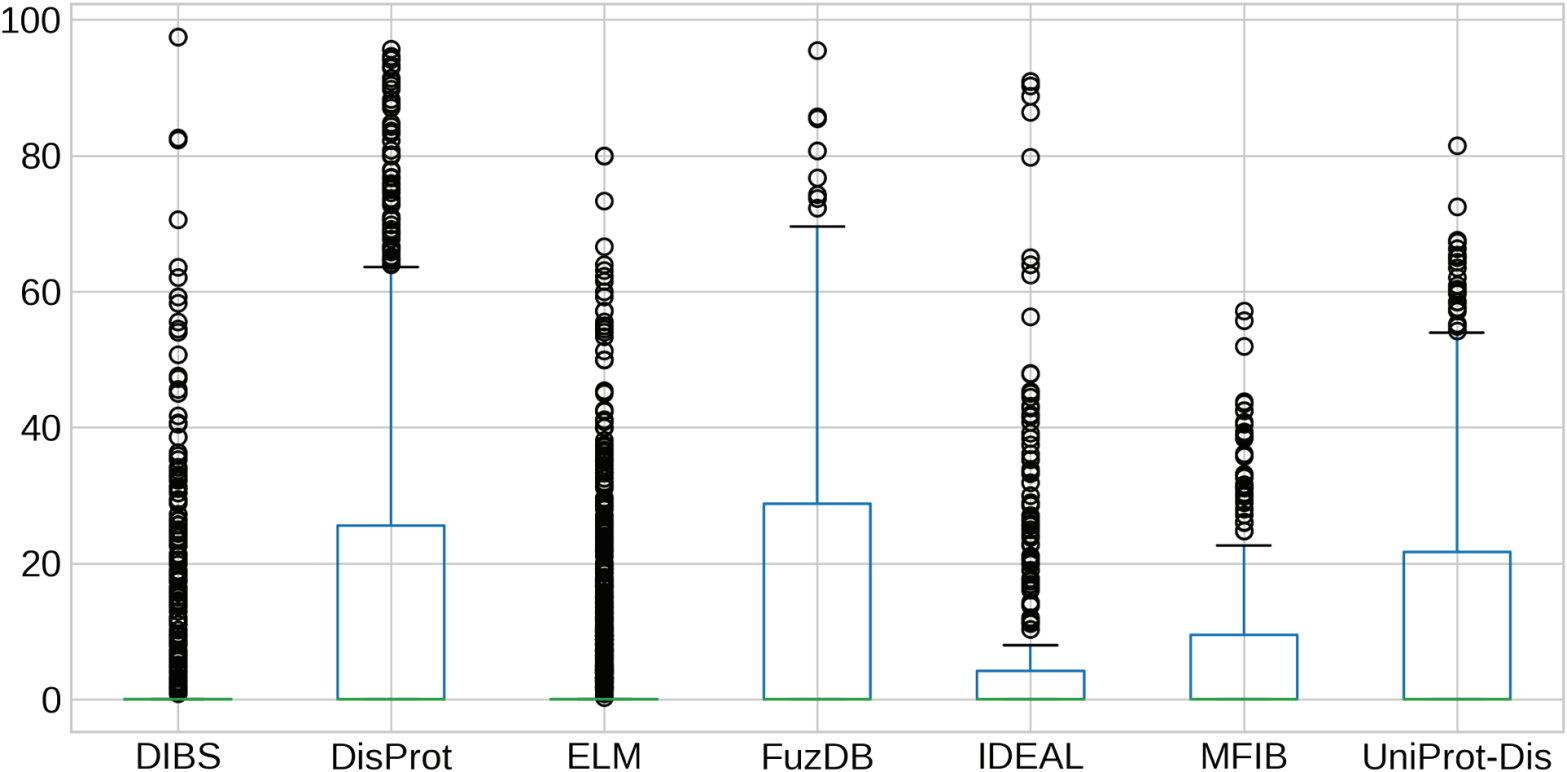
Quantification of LC content of IDRs. The *y*-axis is in logarithmic scale. Box plots of the content of LC per regions in each dataset. Whiskers represent the 25 and 75% of the data and outliers are shown as circles.

### Amino acid frequencies

ID is known to have distinctive amino acid frequencies enriched in charged and hydrophilic amino acids and depleted of hydrophobic ones (10). In order to compare amino acid composition of different ID databases, we calculated the fold increase (or decrease) over the TrEMBL distribution. Enriched and depleted amino acids are shown in Figure 4 as heat map, with databases clustered based on these values. As expected, most hydrophobic amino acids are less frequent. At the other end of the spectrum, negatively charged amino acids (aspartic and glutamic acids) are found more frequently along with positively charged lysine. Some polar amino acids show different behaviors. Serine and glutamine are enriched, while threonine and glutamine are not. Proline is strongly enriched and tryptophan strongly depleted. Figure 4 also highlights some frequencies diverging from the general trend. Cysteine in the UniProt-Disorder dataset is much more frequent and tryptophan, tyrosine and histidine more depleted. Glutamine and asparagine are enriched in FuzDB. Together with DisProt and IDEAL, it is also depleted in arginine, while the remaining datasets are instead enriched. MFIB is slightly enriched in charged residues but still very similar to TrEMBL baseline frequencies. ELM has depletion in alanine and glycine that is more pronounced and proline is more enriched than in the other datasets. Tryptophan is enriched while being depleted in the other datasets.

While absolute frequencies of amino acid distributions of all datasets correlate strongly (Supplementary Figure S6 and 7), the enrichment distribution (fold increase to TrEMBL) highlights significant differences (Supplementary Figure S8). IDEAL, DisProt, DIBS and FuzDB are the most similar, with correlation coefficients ranging from 0.75 (DIBS–FuzDB) to 0.95 (DisProt–IDEAL). UniProt-Disorder, ELM and MFIB correlations to other datasets and between them are not significant. In summary, ELM, UniProt-Disorder and MFIB appear to be the most different databases in terms of amino acid composition, while DisProt, DIBS, FuzDB and IDEAL are very similar.

### Conformational propensity

Das and Pappu proposed a model able to capture ID conformational propensity (tendency to globular/linear structure) by just measuring the fraction of negatively and positively charged residues in an amino acid sequence (27). In doing so, they define some classes within which sequences share a certain structural propensity. We previously applied this classification to ID prediction on the entire UniProt knowledgebase (14). Here, we extend the analysis to compare ID databases (Figure 5). In general, classes 1 to 3 are the most populated. This is expected, since to fall in class 4 or 5 (strong polyelectrolytes) a sequence needs to have a high (>35%) and unbalanced fraction of negatively or positively charged residues. Despite being rarer, instances of classes 4 and 5 are very interesting since they seem to be used mainly as structural components, although in different contexts depending on their net charge. Class 4 (strong negative polyelectrolytes) is employed by eukaryotes in the cytoskeleton, while instances of class 5 (strong positive polyelectrolytes) are found in the ribosome and seem to have been selected to interact with DNA and often display transcriptional activity (14).

DisProt regions are found in all classes, with a strong preference for classes 1–3. A similar distribution is also found in DIBS. ELM shows a radial pattern that is probably an artifact due to the limited range of region lengths, which in turn limits the number of combinations of *f_+_* and *f_−_* values. FuzDB statistics are less significant due to the limited number of regions but otherwise similar to ELM and IDEAL. Both MFIB and UniProt-Disorder are the only two databases significantly different from all other (Supplementary Figure S9) as they show very few cases in classes 4 and 5 and the majority of regions tightly clustered in class 2. UniProt-Disorder in particular shows two clusters, centered on class 2 and shifted toward class 4.

### LC content

Regions of protein sequences with LC are abundant in the protein universe and we wondered about their prevalence in ID databases. As we previously found LC regions more frequently in proteins predicted to be ID with MobiDB-lite (35) than in structured proteins (14), we expected to observe the same overlap in the analyzed datasets. The distribution of the LC content inside IDRs is shown in Figure 6. Surprisingly, the majority of IDRs have a median LC content close to zero. ELM and DIBS regions contain by far the least LC, while DisProt and FuzDB regions are the most enriched and their LC content distributions are not significantly different (Supplementary Figure S10). IDEAL, MFIB and UniProt-Disorder contain less LC than DisProt, with very few regions displaying more than 20% of LC content and they are not statistically different (Supplementary Figure S10).These results are consistent with the notion that ID-binding regions (contained in DIBS, ELM, MFIB and partially in IDEAL) have generally far fewer LC regions than non-binding IDRs (from DisProt, FuzDB and UniProt-Disorder).

### Functional characterization

**Figure 7:**
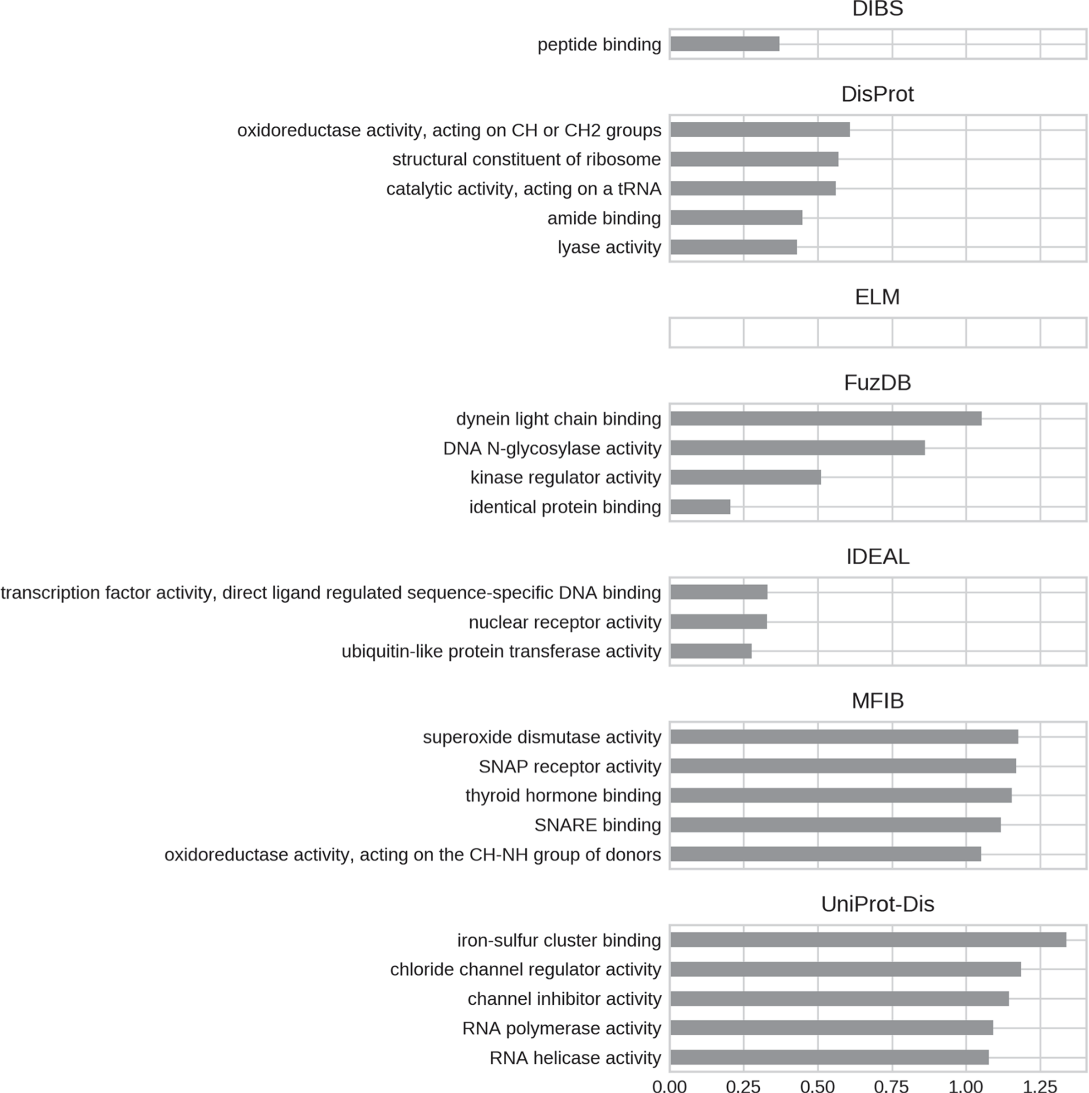
Top five enriched molecular function GO terms. Only high-level terms are shown to improve readability (see Materials and Methods). The *x*-axis represents the fold increase compared to the background.

**Figure 8:**
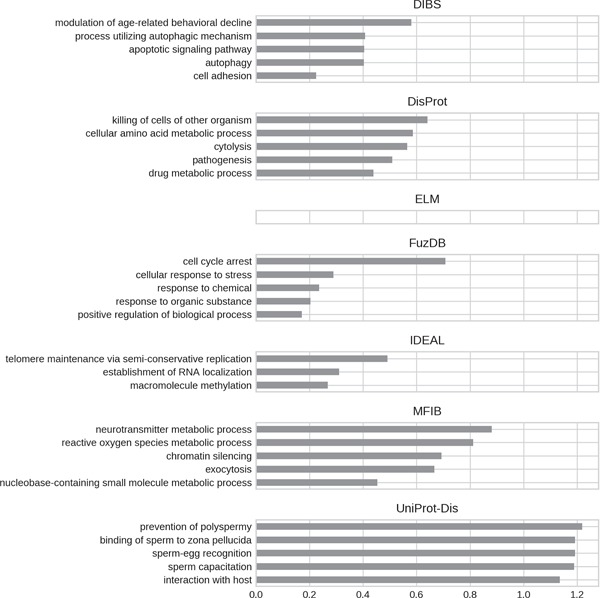
Top five enriched biological process GO terms. Only high-level terms are shown to improve readability (see Materials and Methods). The *x*-axis represents the fold increase compared to the background.

**Figure 9:**
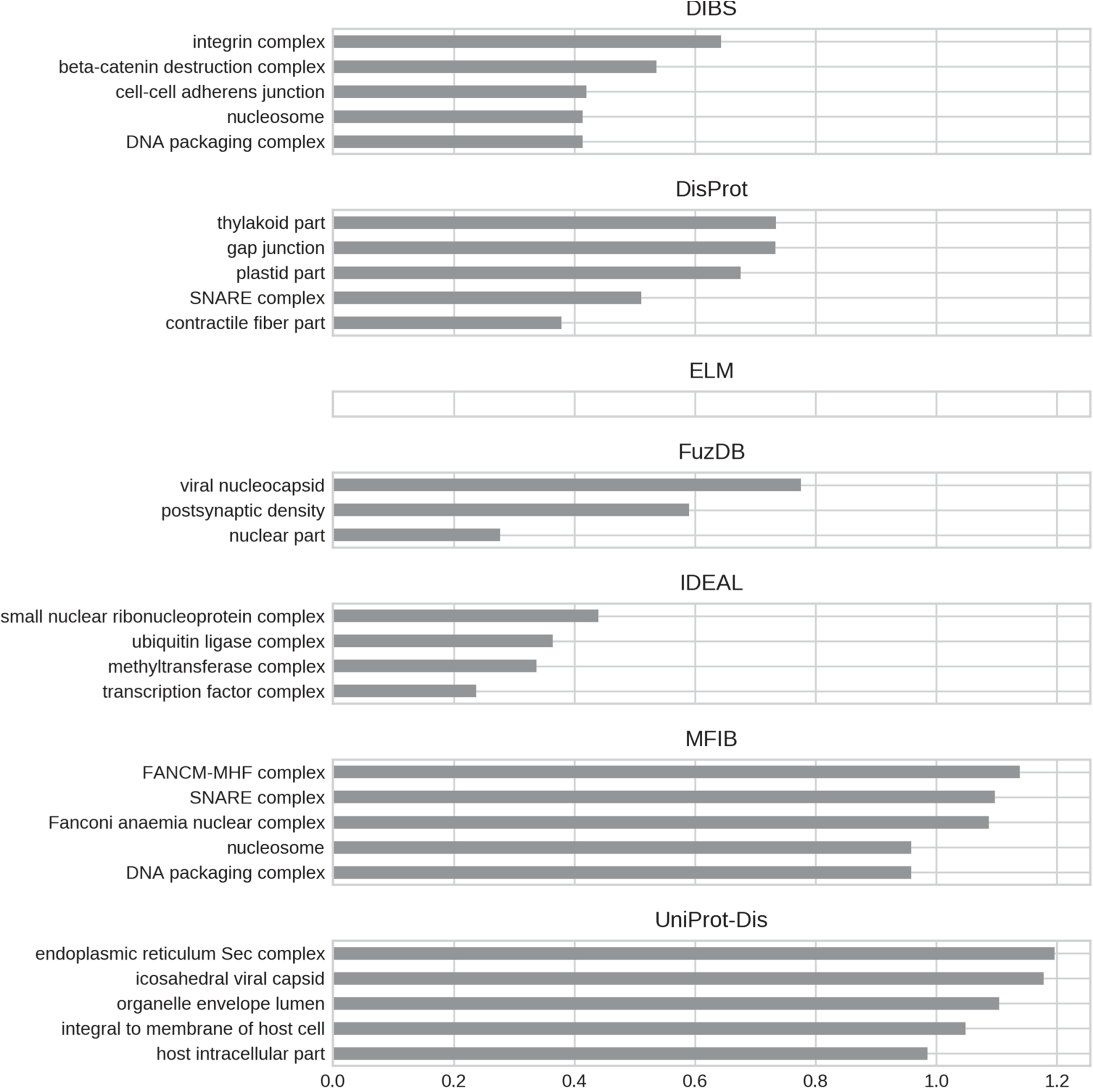
Top five enriched cellular component GO terms. Only high-level terms are shown to improve readability (see Materials and Methods). The *x*-axis represents the fold increase compared to the background.

A final argument compounding the existence of ID flavors may be functional differences. We performed a GO (30) enrichment analysis to test this hypothesis. The five most enriched GO terms (when available) for each ID database are shown in Figures 7–9 for molecular function, biological process and cellular component, respectively. Enrichment was computed considering the union of all datasets as background to highlight the peculiarities of each dataset.

For all datasets, most of GO molecular function terms (Figure 7) are related to binding, which then specializes in the type of interaction partner. DIBS proteins seem to interact mainly with peptides, while IDEAL entries primarily bind DNA and MFIB is enriched in proteins involved in vesicle fusion (e.g. SNAP receptor activity, SNARE binding). Finally, the binding activity enriched in UniProt-Disorder focuses on iron–sulfur cluster binding, which could also explain cysteine enrichment (see Figure 4 and Supplementary Figure S8).We also find the following catalytic activities among ID molecular functions: dismutase activity for MFIB, oxidoreductase activity for DisProt and MFIB and transporter activity for UniProt-Disorder.

GO biological processes (Figure 8) seem to be much more diversified among datasets. DIBS entries focus on cell death and decline. FuzDB is similar but also has terms related to response signaling (response to stress, response to chemical, response to organic substance). As for molecular function IDEAL is enriched in DNA/RNA related terms (telomere maintenance, RNA localization). UniProt-Disorder instead contains terms related to reproduction (prevention of polyspermy, sperm capacitation, sperm–egg recognition). DisProt annotates metabolic related processes, which are also found in MFIB, and pathogen-related terms (killing of cells of other organisms, pathogenesis).

For all GO ontologies, no terms were enriched for the ELM datasets. This means that ELM, which is the largest dataset, covers a wide range of functions without focusing on anyone in particular. Linear motifs are in fact a functionally diverse, all-around strategy (36). DIBS and part of DisProt entries are found in cell–cell junctions. All the databases, with the exception of FuzDB form protein complexes. FuzDB and UniProt-Disorder entries are found in viral capsids or associated to virus activity (endoplasmic reticulum Sec complex).

### Defining ID flavors

The analyzed sources of manually curated ID present many similarities and some significant differences. Each dataset focuses on a particular set of proteins, with surprisingly little overlap between them except for a fraction of entries from DisProt, FuzDB, DIBS and IDEAL. All datasets appear to draw different samples from the large and pervasive population of IDPs. Most annotated proteins come from eukaryotic organisms, although DisProt and MFIB display an interest in bacterial ID, possibly due to experimental biases. UniProt-Disorder focuses on eukaryotes and viruses, the latter being covered by all other datasets to some extent. Very few examples of Archaean IDPs are instead collected. The annotated IDRs have similar lengths, with the exception of ELM annotating short regions by definition and MFIB and UniProt-Disorder which instead collect longer regions. Some differences are highlighted when analyzing the net charge regions. While most regions have balanced and relatively small net charge, IDEAL, DisProt, FuzDB, ELM and DIBS also include regions with high positive or negative net charge. On the other hand, UniProt-Disorder presents very few of these cases and MFIB is completely devoid of them. All datasets follow the typical compositional bias observed in ID, but differences in specific amino acids may reflect different ID flavors. In this study, ID is rarely associated with low sequence complexity, suggesting different functional specializations for ID databases. Concerning the functions, processes and locations on which the ID datasets focus, all of them gather proteins specialized in certain kinds of binding and all include proteins with catalytic activity. However, the processes in which these functionally similar proteins are employed are instead quite diverse, ranging from cell aging and death to reproduction and metabolic processes. The cellular locations enriched in the datasets highlight once again a similarity among different databases (complex and junction) setting apart ELM, which is not enriched by any specific term.

## Conclusions

Our analysis has highlighted how similar, yet different the various ID datasets are. Overall, the main distinction appears between ID-binding regions and general IDRs, in particular, for ELM. It is fair to say that DisProt contains the broadest collection of ID phenomena, with other databases focusing on more specific subsets. FuzDB is largely similar to DisProt, with which it shares half of its entries. Otherwise, the lack of overlap between datasets, while maintaining similar sequence-based features, is reassuring in terms of validity of the approach. It also suggests that there are still many proteins left to be annotated and classified. Curators of ID databases will likely remain busy for years to come.

## Supplementary Material

Supplementary Data
