# Supplementary material for: Where differences resemble: sequence-feature analysis in curated databases of intrinsically disordered proteins
Source: Database (Oxford). 2018 Dec 14;2018:bay127. doi: 10.1093/database/bay127 (PMC6301333; doi:10.1093/database/bay127)
Supplement: Supplementary Data [file bay127_supp.pdf]

Where differences resemble: Sequence-feature analysis in curated databases of intrinsically disordered proteins

Marco Necci[1,2,3], *Damiano Piovesan*[1], *Silvio C.E. Tosatto*[1,4,*]

[1] Dept. of Biomedical Sciences, University of Padua, Padua, Italy
[2] Dept. of Agricultural Sciences, University of Udine, via Palladio 8, 33100, Udine, Italy
[3] Fondazione Edmund Mach, Via E. Mach 1, 38010, S. Michele all'Adige, Italy
[4] CNR Institute of Neuroscience, Padua, Italy
[*] To whom correspondence should be addressed at:
via U. Bassi 58/b, 35131 Padova, Italy
Email: silvio.tosatto@unipd.it; phone: +39 049 827 6269; fax: +39 049 827 6269

Supplementary Material

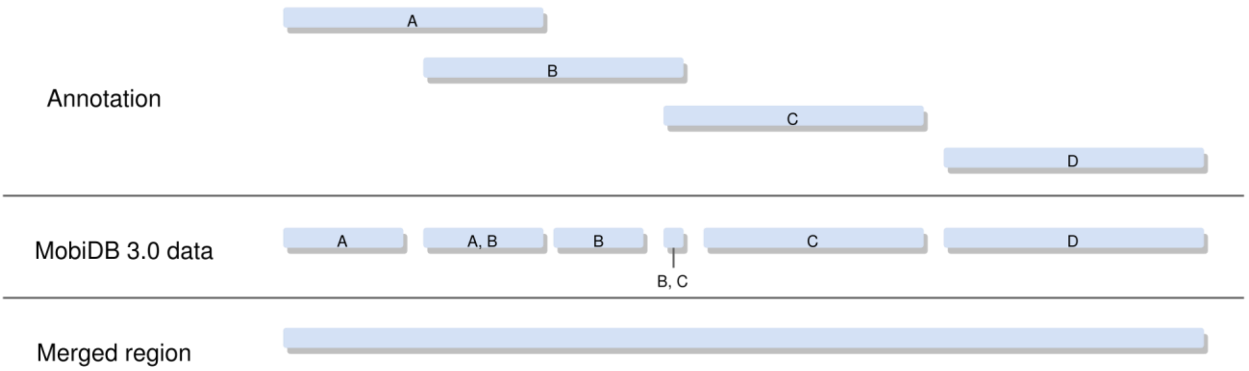

Figure S1 – Merge strategy for overlapping annotation regions. Overlapping annotations in the origin datasets are converted to a series of adjacent annotations in MobiDB 3.0. In this analysis, adjacent annotations are considered as a single annotation.

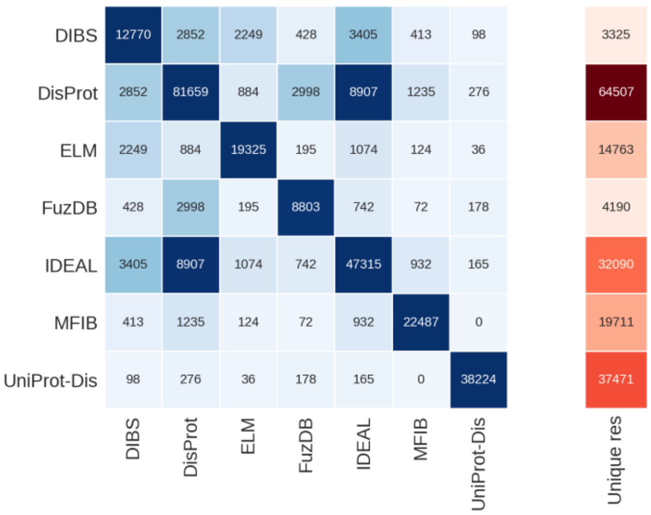

Figure S2 – Number of overlapping residues. Each cell represents the ID regions overlap between a database pair. Unique residues are those annotated uniquely by a single database. The count is by row, e.g. DIBS annotates 3,325 residues.

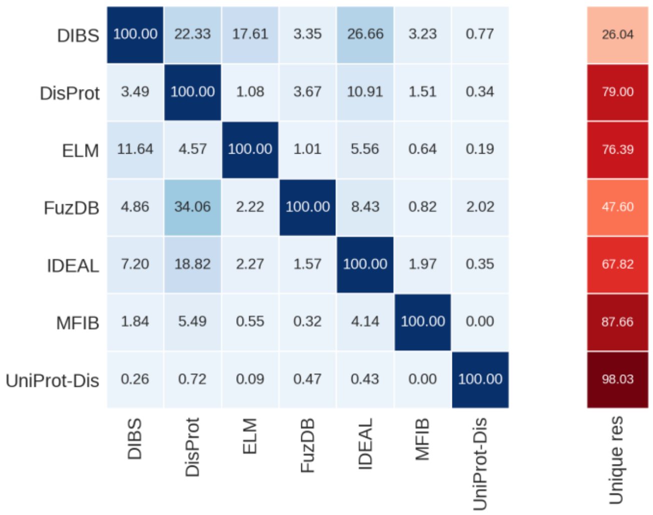

Figure S3 – Fraction of overlapping residues. As in Figure S2 but numbers represent percentages calculated over the database size, e.g. DIBS uniquely annotates 26.04% of its ID residues.

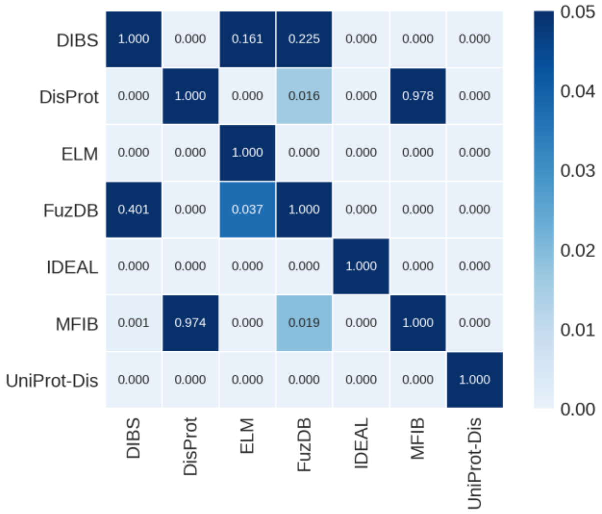

Figure S4 – Taxonomic diversity significance. Significance is calculated with the Chi-square test and represent diversity of the taxonomic distribution, P-value < 0.05 represents significantly different distributions. The Chi-square is directional, but the test is consistent in both directions.

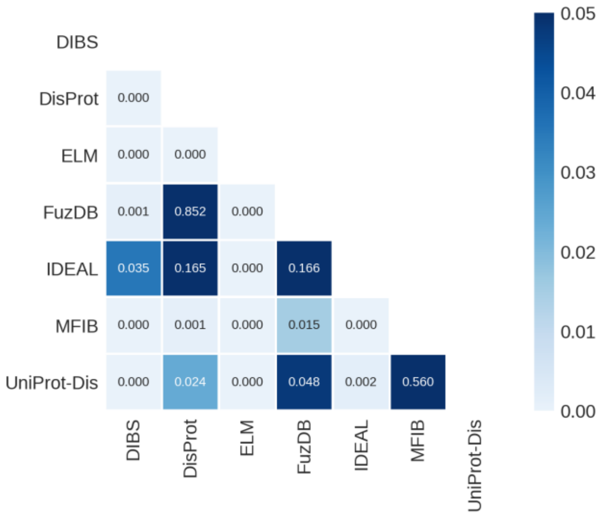

Figure S5 – Heat map of statistical significance of differences in region lengths. The P-value represents the statistical significance of the difference between database length distribution and it is calculate with a T-test.

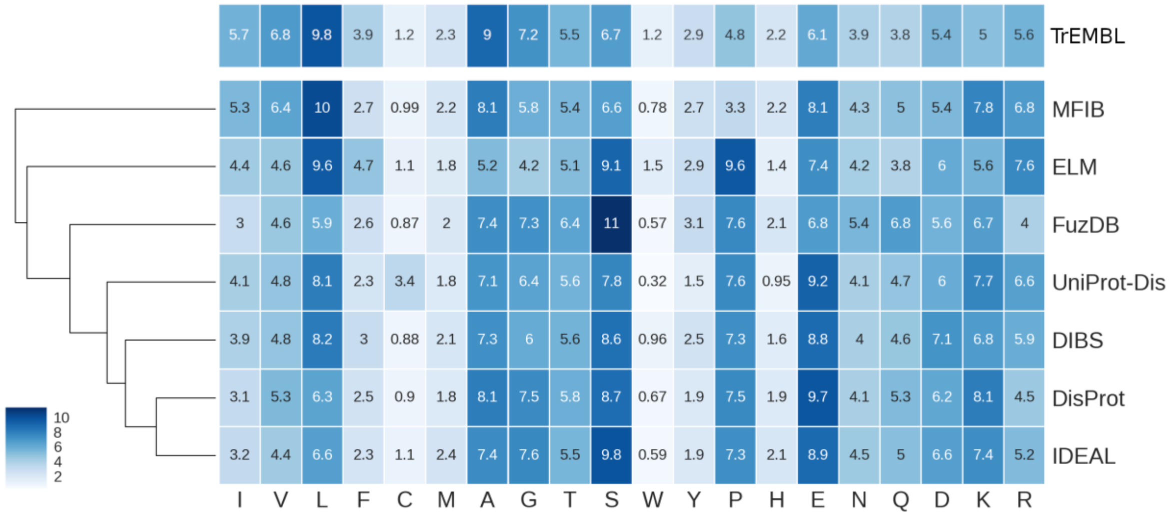

Figure S6 – Hierarchically clustered heat map of amino acid absolute frequencies. Clustering is based on Euclidean distance between frequency vectors. Each value represent amino acid frequencies of each database.

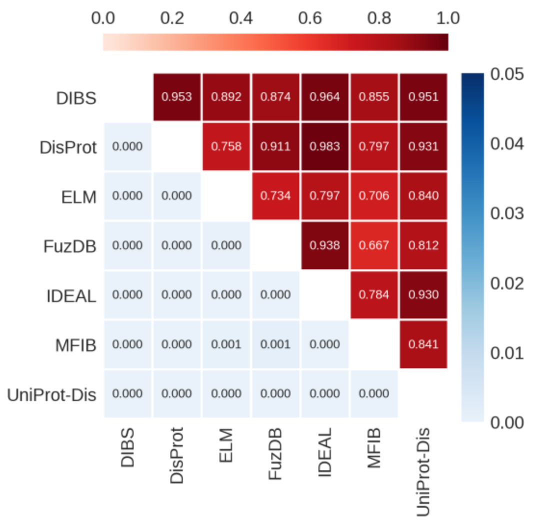

Figure S7 – Correlation matrix of absolute amino acid frequencies. Correlation coefficients (in red) and correlation p-values (in blue) of absolute amino acid.

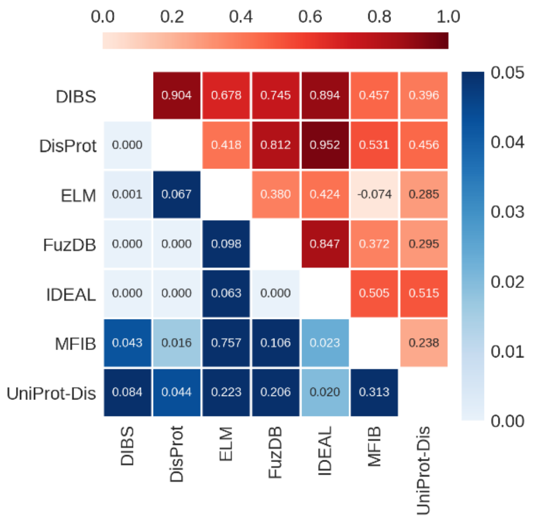

Figure S8 – Correlation matrix of fold increase amino acid frequencies. Correlation coefficients (in red) and correlation p-values (in blue) of fold increase compared to TrEMBL reference frequencies.

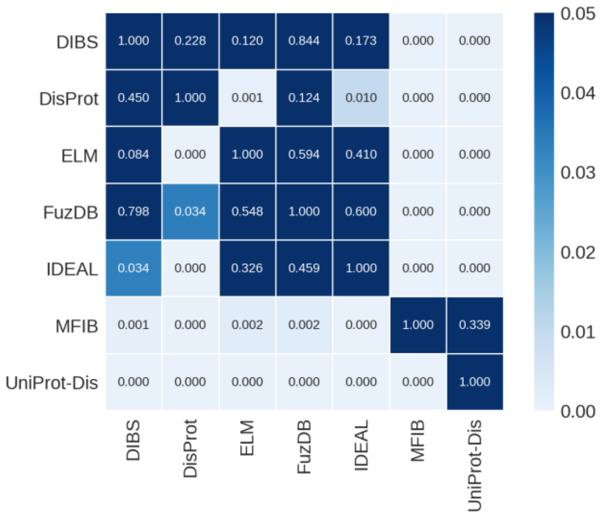

Figure S9 –Heat map of statistical significance of differences in conformational propensity. Significance is calculated with the Chi-square test and represent diversity in the distribution of the five different Pappu's classes. P-value < 0.05 represents significantly different distributions. The Chi-square is directional, but the test is consistent in both directions.

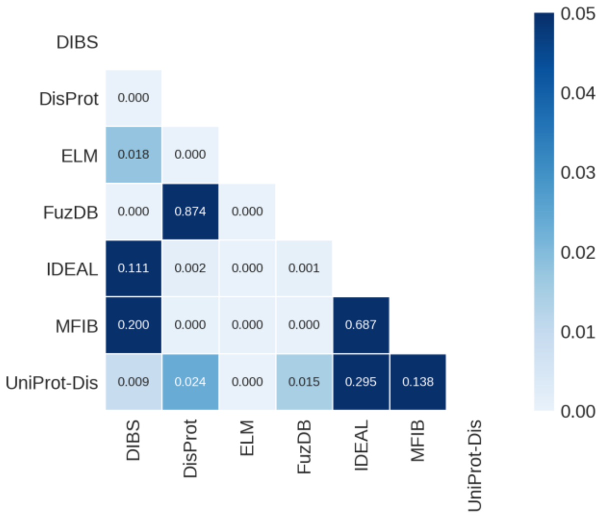

Figure S10 –Heat map of statistical significance of differences in region low complexity content. The P-value represents the statistical significance of the difference between region low complexity content distribution and it is calculate with a T-test.
